# Fox River Valley Medical Association

**Published:** 1864-10

**Authors:** Joseph Tefft, D. W. Young

**Affiliations:** President; Secretary


### Proceedings of Societies.

### FOX RIVER VALLEY MEDICAL ASSOCIATION.

Pursuant to the call issued by the Aurora Medical Association, the Physicians of Aurora and vicinity assembled in the Common Council room, in the city of Aurora, on Thursday afternoon, Sept. 1st, 1864, and, on motion of Dr. Higgins, of Aurora, Dr. Joseph Teft, of Elgin, was called to the Chair, and Dr. D. W. Young, of Aurora, chosen Secretary.

Dr. Young stated the object of the meeting.

Dr. Winchester, of Elgin, moved that the meeting proceed to organize a District Medical Association.

On motion of Dr. Allaire, the Secretary read the following Constitution, which he had drawn up to present to the meeting.

1st. This Association shall be known and distinguished by the name of Fox River Valley Medical Association.

2d. The members of this Association shall collectively represent and have cognizance of the common interests of the Medical Profession in the Fox River Valley; and shall hold their membership by election or invitation.

3d. Any regular practitioner of medicine in good standing in the profession, who holds a diploma granted him by a regularly authorized Medical College, of *legitimate* medicine, or any under graduate, of good moral character, may become a candidate for membership; and if three-fourths of all the members present, at any regular meeting of the Association, vote in his favor, then he shall become a member by signing the Constitution and By-Laws.

4th. Members by invitation shall consist of regular practitioners of medicine of reputable standing in the profession, from any part of the United States. They shall receive an invitation from the Association after an introduction from the member presenting them. They shall hold their connection with the Association until the close of the session at which they are invited, and may participate in the discussions, without the right of voting.

5th. The regular meetings of this Association shall be held on the first Monday of January, April, July, and October of each year. The place of meeting shall be determined for each next succeeding meeting by vote of the Association.

6th. The officers of this Association shall consist of a President, Vice-President, Secretary, Treasurer, and an Executive Committee of three, who shall be elected by ballot, and hold their office for one year, and until their successors are elected.

7th. The President shall preside at the meetings of the Association, preserve order and decorum in debate, give a casting vote when necessary, and perform all such other duties as custom and parliamentary usages may require, and *deliver a good written address* at the expiration of his term of office.

8th. The Vice-President shall assist the President in the performance of his duties, and during the absence, or at the request of the President, shall officiate in his place.

9th. The Secretary shall record the minutes and authenticate the proceedings, conduct the correspondence of the Association, and perform such other duties as the Association may from time to time require.

10th. The Treasurer shall have the immediate charge and management of the funds and property of the Association. He shall report the state of the finances whenever called upon by the Association, and give a full report in writing at the close of his term of office.

11th. The Executive Committee shall also be the Committee on Finance, Credentials, and Printing.

On motion of Dr. Burdick, of Elgin, the above was adopted as the Constitution of this Association.

Dr. Young moved that the gentlemen present now be requested to come forward, sign the Constitution, and become members of this Association.

The following gentlemen then signed the Constitution, and duly become members:—

| | |
|---|---|
| P. A. Allaire, Aurora, Illinois. | O. I. Corbin, Plainfield, Illinois. |
| F. Bartels, Elgin, " | F. N. Burdick, Elgin, " |
| J. Nicoloy, Plainfield, " | E. J. Morse, Naperville, " |
| S. F. Hance, Aurora, " | Joseph Teft, Elgin, " |
| O. D. Howell, Aurora, " | S. O. Long, Big Rock, " |
| S. C. Gillett, Aurora, " | N. F. Eddy, Geneva, " |
| E. Winchester, Elgin, " | Wm. LeBaron, Geneva, " |
| L. H. Angell, Aurora, " | L. A. Winslow, Aurora, " |
| D. S. Jenks, Plano, " | George Higgins, Aurora, " |
| C. Cushing, Warrenville, " | D. W. Young, Aurora, " |

The Secretary, Dr. Young, then read the following By-Laws of the Aurora City Medical Association:—

The order of business at the regular meetings of this Association shall, at all times, be subject to the vote of three-fourths of all the members in attendance; and until permanently altered, except when for a time suspended, it shall be as follows:—

1. Calling the meeting to order.
2. Calling the roll of officers.
3. Reading the minutes of last meeting.
4. Reception of members by invitation.
5. Proposals for membership.
6. Unfinished business.
7. New business.
8. Reading and dicussing voluntary communications.
9. Correspondence.
10. Selection of next place of meeting.
11. Adjournment.

Members of this Association shall be liable to censure, suspension, repremanding, or expulsion for wilful neglect, or disregard of the rules and regulations of this Association, or violation of our code of professional ethics. A vote of two-thirds of the members present shall be required to censure or suspend, and one of three-fourths to expel.

In case of any charges being preferred against any member, which might lead to his censure, suspension, or expulsion, the Association shall immediately give to the accused a written copy of the charges preferred. The matter shall then lie over till the next regular meeting, when due action shall be taken thereon.

Five members shall constitute a quorum; a less number may adjourn.

On motion of Dr. Young, the Code of Medical Ethics adopted by the American Medical Association were adopted for the government of this Association.

On motion of Dr. Howell, the above By-Laws were adopted as the By-Laws of this Association.

On motion of Dr. Winslow, the Association then proceeded to the election of officers for the ensuing year.

On motion of Dr. Allaire, Dr. S. F. Hance, of Aurcra, and Dr. S. O. Long, of Big Rock, were appointed tellers.

The balloting resulted as follows:—

President, Joseph Tefft, M.D., of Elgin.

Vice-President, P. A. Allaire, M.D., of Aurora.

Secretary, D. W. Young, M.D., of Aurora.

Treasurer, Wm. LeBaron, M.D., of Geneva.

Executive Committee, O. I. Corbin, M.D., of Plainfield; O. D. Howell, M.D., of Aurora, and S. O. Long, M.D., of Big Rock.

On motion of Dr. L. H. Angell, the above were declared duly elected as the officers of this Association for the ensuing year.

Dr. Winchester, of Elgin, suggested that the Association ought to adopt a fee bill, so that we would have a uniform rate of charges throughout the entire district.

By request the Secretary read a portion of the fee bill adopted by the Aurora City Medical Association in April, 1857.

It was the unanimous opinion of the meeting that, in consequence of the increased price of all kinds of drugs and medicines, and the increased expenses of living, these rates are *too low* for these times.

Dr. Allaire moved that the rates be increased to two dollars per visit in the cities, for day visits.

Dr. Winchester moved to amend by inserting $1.50 instead of $2. Amendment prevailed, and the following rates were agreed upon.

For each visit and medicine in city, during the day time, $1.50; for each visit and medicine in the city, in the night, $2.50.

*Country Business.*—One mile, $1.50; thereafter add 50 cents per mile for day time; nights add 25 per cent.

For each obstetric case in city, $7.50; in country add traveling fee 50 cents per mile after the first mile.

For examination and medicine at house or office, from $1.00 to 10.00.

On motion of Dr. Winchester, of Elgin, Drs. Young, Allaire, and Winslow, of Aurora, were appointed a Committee to revise the balance of the fee bill.

On motion of Dr. Allaire, the Association adjourned, to meet at the Common Council Room, at Aurora, on the first Monday of October, next, at one o'clock P.M.

JOSEPH TEFFT, M.D., *President.*

D. W. YOUNG, M.D., *Secretary.*

